# Upcycling of polyurethane waste by mechanochemistry: synthesis of N-doped porous carbon materials for supercapacitor applications

**DOI:** 10.3762/bjnano.10.157

**Published:** 2019-08-06

**Authors:** Christina Schneidermann, Pascal Otto, Desirée Leistenschneider, Sven Grätz, Claudia Eßbach, Lars Borchardt

**Affiliations:** 1 Technische Universität Dresden, Department of Inorganic Chemistry, Bergstraße 66, 01069 Dresden, Germany; 2 University of Alberta, Department of Chemical and Materials Engineering, 12-340 Donadeo Innovation Centre for Engineering, 9211 - 116 Street, AB T6G 1H9 Edmonton, Canada; 3 Ruhr-Universität Bochum, Department of Inorganic Chemistry, Universitätsstrasse 150, 44801 Bochum, Germany

**Keywords:** mechanochemistry, polyurethane, porous carbon, supercapacitor, waste

## Abstract

We developed an upcycling process of polyurethane obtaining porous nitrogen-doped carbon materials that were applied in supercapacitor electrodes. In detail, a mechanochemical solvent-free one-pot synthesis is used and combined with a thermal treatment. Polyurethane is an ideal precursor already containing nitrogen in its backbone, yielding nitrogen-doped porous carbon materials with N content values of 1–8 wt %, high specific surface area values of up to 2150 m^2^·g^−1^ (at a N content of 1.6 wt %) and large pore volume values of up to 0.9 cm^3^·g^−1^. The materials were tested as electrodes for supercapacitors in aqueous 1 M Li_2_SO_4_ electrolyte (100 F·g^−1^), organic 1 M TEA-BF_4_ (ACN, 83 F·g^−1^) and EMIM-BF_4_ (70 F·g^−1^).

## Introduction

Currently more than 275 million tons of plastics end up as waste every year, 12.7 million tons of which accumulate in the oceans [1–2]. This waste is mainly packaging materials such as polyethylene (PE), polypropylene (PP), polyurethane (PU), disposable bottles such as polyethylene terephthalate (PET) and construction materials such as polyvinyl chloride (PVC) and PU. At the same time, more than 300 million tons of new plastic materials are produced every year, with an increasing tendency [3–5]. So far, different recycling techniques have been devised to counteract environmental pollution through accumulation of plastic waste. Especially, the recovery of PE and PP, as well as depolymerization processes for PET and the reprocessing of PVC by crushing and melting in conversion systems are well developed [6–9]. Commonly, 10–30% of the plastic waste is recycled by manufacturing new plastic products. Another 10–25% is used for energy recovery as fuel for industrial processes. However, 55–80% still end up in landfills or even in the environment [3–4,10]. Some of the polymers that accumulate as plastic waste are poorly recyclable because of low recycling yields and insufficient properties of the recycled polymers in terms of elasticity, rheology, and thermal and mechanical stability [5]. Amongst them is PU, a thermosetting polymer with a cross-linked structure [5,11–12]. PU is mainly used for the production of disposable packaging materials and sponges, for long-term applications in upholstered furniture and car seats, and as spray foam for insulation [13–15]. Approximately 19 million tons of PU waste accumulate annually [13,15–18]. Therefore, it is essential to develop sustainable upcycling methods that reduce environmental pollution on the one hand and ensure a good material utilization on the other hand. One approach is the synthesis of porous carbon materials from PU waste. At the industrial scale, activated carbon materials are already obtained from coconut shells and other biomass waste [19–21]. However, the industrial use of plastics for this purpose has not been established yet.

The main properties of porous carbon materials [22–23] such as high specific surface area and high electrical conductivity allow for a variety of applications in catalysis [24–26], gas sorption/separation [27–29] and electrochemical energy storage/conversion. For the latter, porous carbon materials are established as electrode materials in fuel cells [30–33], Li–S cells [34–37], and supercapacitors [38]. In addition, these carbon materials can be functionalized with heteroatoms such as nitrogen, which was reported to affect the electrical conductivity [39–42], the energy storage capacity, and the wettability of the electrodes with electrolyte [43–45]. Commonly, nitrogen is inserted into the carbon framework either by solution-based impregnation with nitrogen-containing precursors, e.g., melamine or urea [19,46], or via post-treatment processes with gaseous, nitrogen-containing precursors, e.g., N_2_ or NH_3_, at high temperatures [31,47–48]. In PU nitrogen is already part of the urethane group rendering it a suitable nitrogenous carbon precursor. The conventional production of N-doped porous carbon materials from PU, however, requires many process steps and produces large quantities of solvent waste from crushing and dissolving steps, the addition of toxic chemicals, as well as the subsequent drying and carbonization [49]. The utilization of a solvent in general has to be critically examined, since it has to be separated from the product, which is time-consuming and costly, and later on accumulates as waste that has to be reprocessed in an energy-intensive procedure. Furthermore, solvents can be hazardous to the environment or toxic to humans. PU, for example, is hardly soluble and some PU materials only dissolve in organic solvents such as DMF, DMSO or THF. Consequently, it is necessary to implement sustainable and effective processes that use renewable raw materials or plastic waste and can be conducted in a solvent-free manner [50–52]. Mechanochemistry is an innovative synthesis concept that can be conducted without solvents. It is cost-efficient and sustainable at the same time [53]. Mechanochemistry is well established in the field of pharmaceutical [54–55], organic [56–58], and inorganic chemistry [59–62]. Mechanochemical reactions are initiated and controlled by mechanical energy, for example provided by the collisions of milling balls in high-energy ball mills. The advantages of mechanochemistry are obvious. Syntheses can be conducted without solvents [63–64], and within short reaction times [59,65]. Also, the potential of mechanochemistry for upscaling has recently been discussed by Stolle and co-workers. An upscaling from the milligram scale to the multiple-gram scale has been shown to be feasible [66–67]. For the kilogram scale other milling techniques such as impact mills or extruders may be applicable [68].

**Figure 1:**
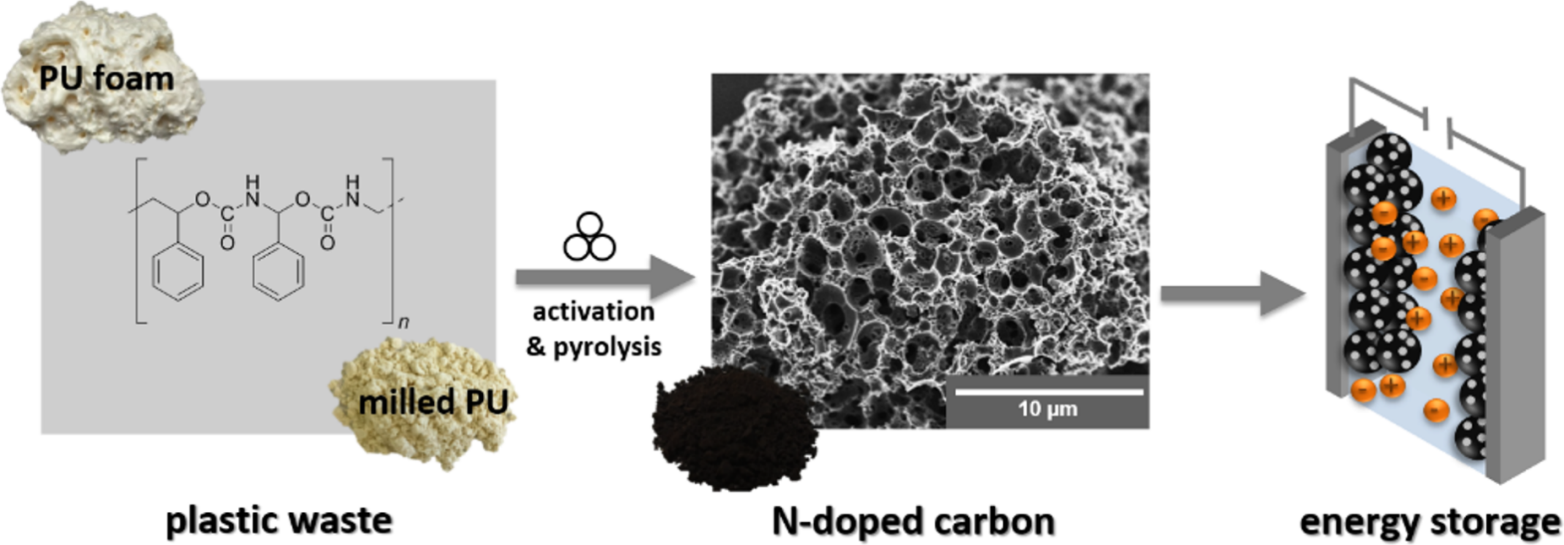
Upcycling approach consisting of high-energy ball milling and carbonization of a mixture of PU foam as the carbon source and potassium carbonate (K_2_CO_3_) as an activation reagent to form nitrogen-doped porous carbon as an electrode material for supercapacitors.

Here, we present a fast and scalable synthesis for the production of N-doped carbon materials (Figure 1). PU (spray foam) is used as a nitrogenous carbon source and potassium carbonate (K_2_CO_3_) is used as an activation agent. Urea (CH_4_N_2_O) can optionally be added to further increase the nitrogen content. Pre-milling of the PU foam and the mechanochemical reaction of all components are carried out in a planetary ball mill. The received polymer mixture is carbonized to form a nitrogen-doped carbon material with a surface area of up to 2150 m^2^·g^−1^ and a total pore volume of up to 0.9 cm^3^·g^−1^. In order to generate different nitrogen contents and to increase the porosity of the carbon material, we used different ratios of urea and K_2_CO_3_. Moreover, the N-doped carbon materials have been investigated as electrode material for supercapacitors in aqueous Li_2_SO_4_, organic TEA-BF_4_ in acetonitrile, and an ionic liquid EMIM-BF_4_ electrolyte.

## Results and Discussion

### Characterization and mechanochemical treatment of PU

Polyurethane is a polymer formed by polyaddition of diisocyanates R^1^(–NCO)_2_ with polyols R^2^(–OH)*_n_*. It is characterized by the resulting urethane group NH-(CO)-O. The special feature of these compounds is the large variety of monomers that can be used for the production of polyurethane materials, such as various aliphatic or aromatic isocyanate components (R^1^) and dihydric or polyhydric alcohols as polyol components (R^2^). This wide range of monomers allows for different functional groups to be integrated into the PU framework and the adjustment of certain reaction conditions or specific properties, such as ensuring fast reactions and stable polymer chains through aromatic isocyanates. The spray foam used here (PU-F) is a one-component foam and, according to the supplier (Soudal), consists of polyisocyanate with an aromatic residual group (polymethylene–polyphenyl isocyanate). Due to the delocalized charges in the aromatic residue of the monomers, they directly react with moisture and do not require an additional diol, as is the case with two-component foams. The spray of the one-component foam contains small amounts of a flame retardant (tris(2-chloro-1-methylethyl)phosphate) and propellants such as propane, isobutene and dimethyl ether, which cause the foaming. The foam has a low density of 25 kg·m^−3^, which is required for the application, but makes recycling difficult due to its poor processability, and low yields of energy and material. High-energy ball milling is initially used for comminution of the foam (PU-F) to a powder (PU-BM). This does not lead to any changes in the chemical bonding according to infrared spectra (Figure S1A, Supporting Information File 1).

**Figure 2:**
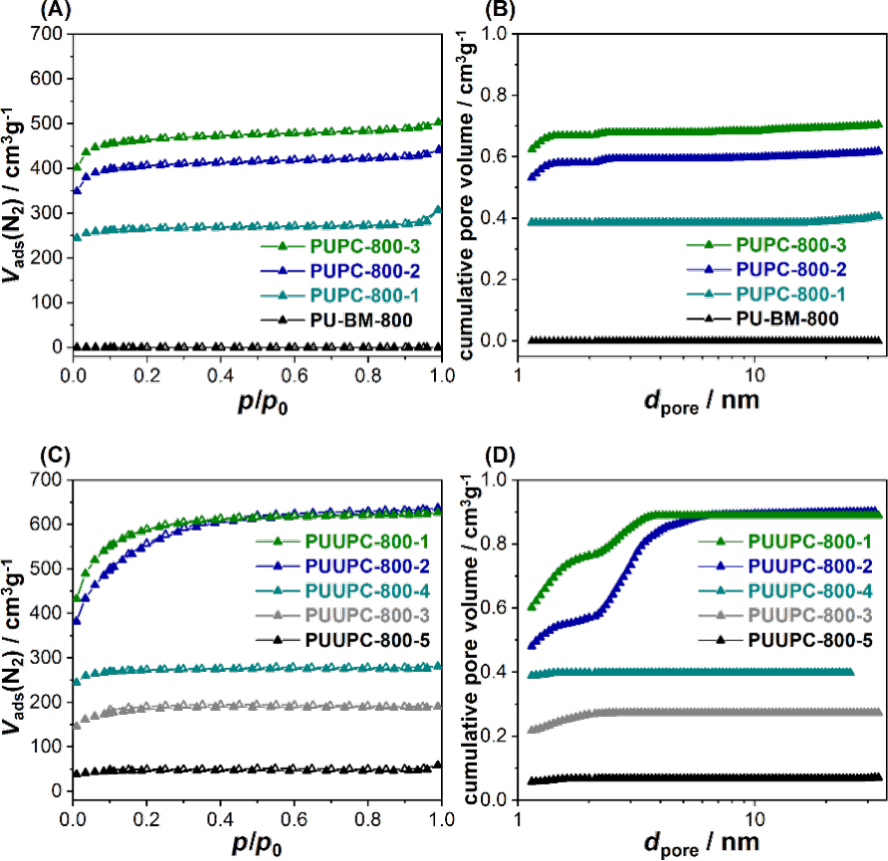
(A, C) Nitrogen adsorption/desorption (filled symbols/empty symbols) isotherms (measured at −196 °C) and (B, D) cumulative pore size distribution (PSD) using QSDFT with cylindrical/slit-shaped pores of the samples (A, B): PUPC-800-1 (cyan), PUPC-800-2 (blue), PUPC-800-3 (green), and PU-BM-800 (black) and the samples (C, D): PUUPC-1-800 (green), PUUPC-2-800 (blue), PUUPC-3-800 (grey), PUUPC-4-800 (cyan) and PUUPC-5-800 (black).

After addition of K_2_CO_3_ powder, the mechanochemical treatment also ensures a homogeneous distribution of the latter within the polymer. This ensures an optimal subsequent activation process. First investigations were made to understand the influence of the K_2_CO_3_ concentration on the activation process by varying the K_2_CO_3_ content, while keeping the PU content constant (Table 1). The samples are indexed as follows: polyurethane (PU), K_2_CO_3_ (PC), and “800” standing for the pyrolysis temperature and a sequence number at the end of the sample code. The obtained plastic-derived carbon materials were investigated by N_2_ physisorption at −196 °C (Figure 2A), and the calculation of the pore size distributions was carried out under the assumption of slit and cylindrical pore geometry using quenched solid density functional theory (QSDFT; Figure 2B).

**Table 1:** Characterization data of different N-doped carbon samples after milling and carbonization. Physisorption data derived from N_2_ isotherms measured at −196 °C. Element concentrations derived from elemental analysis.

sample	mass ratio of PU/urea/K_2_CO_3_	SSA_BET_^a^ / m^2^·g^−1^	SSA_DFT_^b^ / m^2^·g^−1^	*V*(N_2_)_total_^c^ / cm^3^·g^−1^	*V*(N_2_)_micro_^d^ / cm^3^·g^−1^	*V*(N_2_)_meso_^e^ / cm^3^·g^−1^	*W_x_*^f^ / wt %			

							N	C	H	rest

PU-BM	—	0.2	0	—	—	—	5.7	61.0	6.3	27
PU-BM- 800	—	0	0	—	—	—	3.9	81.7	0.8	13.6
PUPC- 800-1	3:0:1	950	1383	0.41	0.39	0.02	1.1	86.2	0.3	12.4
PUPC- 800-2	3:0:2	1421	1953	0.62	0.58	0.04	1.1	71	1.2	26.7
PUPC- 800-3	3:0:3	1668	2094	0.71	0.67	0.04	1.2	61.8	0.9	36.1
PUUPC- 800-1	3:1:3	2147	2029	0.89	0.76	0.13	1.6	62.0	1.0	35.4
PUUPC- 800-2	3:2:3	2005	1390	0.84	0.61	0.23	2.8	60.0	1.2	36.0
PUUPC- 800-3	3:3:3	668	823	0.27	0.27	—	6.3	64.4	1.9	27.4
PUUPC- 800-4	3:3:2	1005	1368	0.40	0.40	—	7.4	62.0	2.7	27.9
PUUPC- 800-5	3:3:1	174	221	0.07	0.07	—	8.4	63.3	1.8	26.5

^a^Multi-point BET-method for 0.05 ≤ *p*/*p*_0_ ≤ 0.2; ^b^SSA of micropores determined by QSDFT below 2 nm; ^c^total pore volume at *p*/*p*_0_ = 0.95; ^d^applying QSDFT method assuming slit and cylindrical shaped pores using the adsorption branch; ^d^*V*_pore,meso_ = *V*_pore,total_ − *V*_pore,micro_; ^f^chemical composition (*W_x_*) obtained from elemental analysis, rest refers to the residual mass supposed to be oxygen, which is undetectable with this methode.

The sample PU-BM-800 (i.e., the reference with no K_2_CO_3_) does not show any porosity and a slightly decreased nitrogen content of 3.9 wt % compared to the pure PU-BM (5.7 wt %, Table 1). In contrast, a microporous material is obtained after adding only a small amount of K_2_CO_3_ (PUPC-800-1, Figure 2A) [69]. PUPC-800-1 exhibits a surface area of 950 m^2^·g^−1^, a total pore volume of 0.41 cm^3^·g^−1^ and a N content of 1.1 wt %. Please note, the nitrogen content decreased during the activation process and thus nitrogenous compounds must have been released from the polymer during carbonization. The further increase of the K_2_CO_3_ content results in even more porous carbon materials with increased surface area of 1420 m^2^·g^−1^ (PUPC-800-2) and 1670 m^2^·g^−1^ (PUPC-800-3) and pore volume of 0.62 cm^3^·g^−1^ and 0.71 cm^3^·g^−1^, while the N content does not further decrease and remains at 1.1 wt % and 1.2 wt %, respectively (Table 1). The proposed mechanism according to McKee et al. is given in section S3 of Supporting Information File 1 [70]. It can be concluded, that the activation of PU foam provides porous carbon materials, but the activation alone is not sufficient to ensure both, a high surface area and nitrogen content.

### Increase of the N content through addition of urea

Since we observed a decreasing N content during the activation process of PU, we added urea as an additional nitrogenous precursor to the mechanochemical synthesis. Please note the modified sample code, with polyurethane–urea–K_2_CO_3_ as PUUPC and a new sequence number (Table 1). The IR spectra reveal that polymerization reactions occur during ball milling (Figure S1B, Supporting Information File 1). In particular, the condensation of urea with the urethane group is initiated during the milling process shown by the disappearing peaks of NH_2_ (3335 cm^−1^) and of C=O (1715 cm^−1^). In addition, the morphological conversion of the PU powder (Figure S3, Supporting Information File 1) to an agglomerated polymer (Figure S4, Supporting Information File 1) as well as the optical observation that moisture is produced during milling, indicates that a condensation reaction must have taken place.

Since an equal ratio of PU and K_2_CO_3_ (PUPC-800-3) has yielded the highest porosity in the previous section, we kept the PU and K_2_CO_3_ ratio constant and added specific amounts of urea to investigate the influence of the nitrogen precursor on the nitrogen content and surface area. Already by adding small amounts of urea, the nitrogen content of the obtained N-doped carbon materials is slightly increased to 1.6 wt % (PUUPC-800-1) and 2.8 wt % (PUUPC-800-2, Table 1). Moreover, the addition of urea influences the activation process itself [64], resulting in an increased surface area of 2150 m^2^·g^−1^ (PUUPC-800-1) and 2010 m^2^·g^−1^ (PUUPC-800-2) and pore volume of 0.89 cm^3^·g^−1^ and 0.84 cm^3^·g^−1^ with a small fraction of mesopores (Figure 2C,D) [69]. However, when urea was added in the same ratio as PU and K_2_CO_3_ (PUUPC-800-3), the surface area and the pore volume significantly decreased to 670 m^2^·g^−1^ and to 0.27 cm^3^·g^−1^, while the nitrogen content increases to 6.3 wt % (Table 1). This observation is related to an intensified chemical activation process due to the high amount of urea and the formation of ammonia during the high-temperature treatment, leading to a higher consumption of carbon and its partial textural destruction. In addition to the formation of ammonia and the activation of the carbon, urea can form (NH_4_)_2_(CO_3_), which further decomposes to gaseous H_2_O, CO_2_ and NH_3_ and leads to additional porosity of the carbon.

In order to attenuate the activation process, while ensuring a high nitrogen content at the same time, we reduced the K_2_CO_3_ content, while keeping the content of PU and urea constant. Reducing the K_2_CO_3_ content results in a specific surface area of 1010 m^2^·g^−1^, a nitrogen content of 7.4 wt % and a pore volume of 0.40 cm^3^·g^−1^ (PUUPC-800-4, Table 1). However, further reduction of the K_2_CO_3_ content leads to a decreased porosity (SSA = 170 m^2^·g^−1^), while the N content is further increased up to 8.4 wt % (PUUPC-800-5, Table 1). As a result, if the K_2_CO_3_ content is insufficient, the activation process is incomplete and a high porosity cannot be obtained.

**Figure 3:**
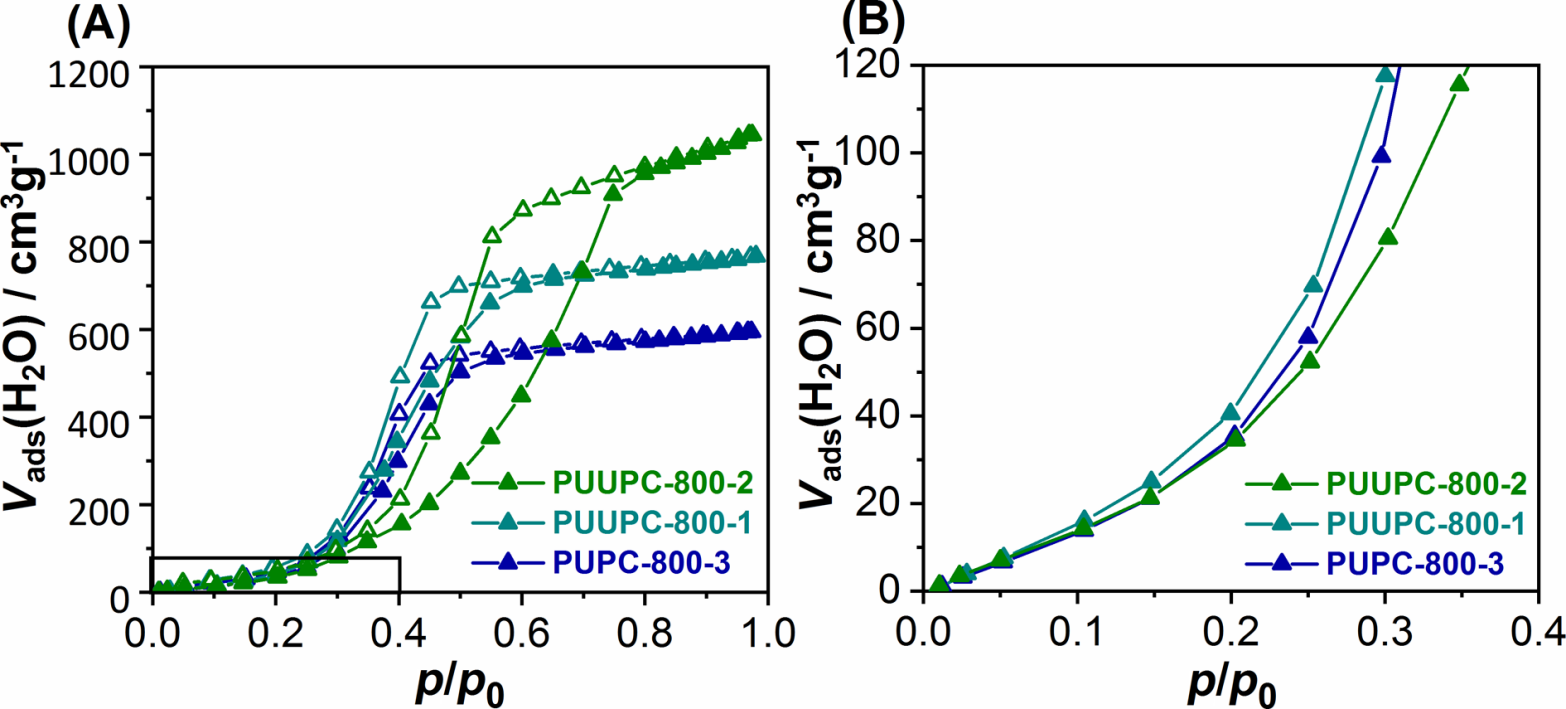
(A) Water vapor sorption isotherms (adsorption/desorption = filled symbols/empty symbols) measured at 25 °C and (B) magnification of the lower relative pressure range of the samples: PUUPC-800-1 (cyan), PUUPC-800-2 (green) and PUPC-800-3 (blue).

Water vapor adsorption was performed exemplarily for the samples PUPC-800-3, PUUPC-800-1, and PUUPC-800-2 to demonstrate the effect of the porosity and the generated nitrogen functionalities on sorption, phase and wetting behavior. The water isotherms of all measured samples are assigned to a type V isotherm according to the IUPAC classification (Figure 3A) [69]. Up to a relative pressure of *p*/*p*_0_ < 0.4 almost no adsorptive interactions take place. The step at a relative pressure of *p*/*p*_0_ = 0.4 is assigned to the filling of micropores. PUUP-800-1 shows the highest uptake in this range because it has the highest micropore volume. At relative pressures of *p*/*p*_0_ > 0.8, PUUP-800-1 shows a lower water adsorption uptake than sample PUUP-800-2. This can be attributed to the higher mesopore volume of PUUP-800-2 [71]. A direct correlation between the nitrogen content of the samples and the water adsorption behavior is not observed since the uptake is not significantly shifted to lower relative pressures. A reduction of the total amount of adsorbed water is observed for sample PUPC-800-3 and corresponds to a decreased total pore volume and nitrogen content (Table 1, Figure 2B,D). The hydrophilicity of the N-doped carbon materials has been confirmed by using the dynamic contact angle technique (Figure S7, Supporting Information File 1). As expected, the samples absorbed the water droplet almost immediately after its release. The sample PUUPC-800-2 absorbed the water droplet completely after only 8 s, whereas sample PUPC-800-3, exhibiting a lower nitrogen content, absorbed the water after 20 s. Thus, the higher nitrogen content benefits the wettability of the carbon surface.

### Electrochemical characterization

The produced carbon materials differ in terms of specific surface area, pore sizes, and nitrogen content. Therefore, we selected three carbon materials that represent a wide range of structure characteristics for electrochemical characterization: PUPC-800-3, PUUPC-800-1, and PUUPC-800-2. First, we determined the powder resistance of the carbon materials. Two general trends are observable. Firstly, the resistance increases with a higher specific surface area (compare PUUPC-800-1 and PUPC-800-3, Table 2). This was previously observed by Casco et al. [39] for a different carbon system, too. Secondly, regarding PUUPC-800-1 and PUUPC-800-2, which mainly differ in their nitrogen content, the resistance increased with increasing nitrogen content. Thus, nitrogen-doping has no beneficial influence on the conductivity of the electrodes in contrast to expectations.

**Table 2:** Electrochemical characterization data of PUPC-800-3, PUUPC-800-1 and PUUPC-800-2 measured in aqueous (1 M Li_2_SO_4_), organic (1 M TEA-BF_4_ in ACN) and ionic liquid (EMIM-BF_4_) electrolytes calculated from galvanostatic charge–discharge measurements at different specific currents.

electrolyte	powder resistance^a^ / Ω·cm	specific current / A·g^−1^	specific capacitance^b^ / F·g^−1^		

			1 M Li_2_SO_4_	1 M TEA-BF_4_(ACN)	EMIM-BF_4_

PUPC-800-3	0.39	0.1	90	47	56
		1	73	62	71
PUUPC-800-1	0.47	0.1	90	73	69
		1	81	72	63
PUUPC-800-2	0.75	0.1	99	59	36
		1	82	57	42

^a^Powder pressed with 2 t, *d* = 1 cm; ^b^obtained from the discharge branch.

**Figure 4:**
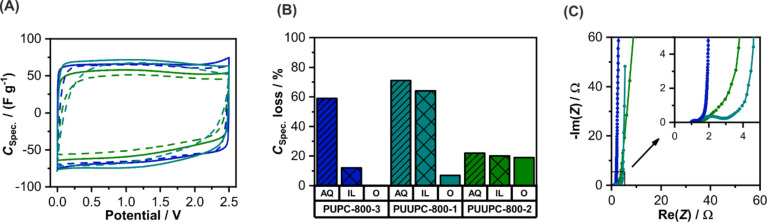
(A) Cyclic voltammogram measured in 1 M TEABF_4_ (ACN) with a scan rate of 10 mV·s^−1^ (solid lines) and 100 mV·s^−1^ (dashed lines), (B) loss of the specific capacitance in three different electrolytes, and (C) Nyquist plot for the three samples PUPC-800-3 (blue), PUUPC-800-1 (cyan), and PUUPC-800-2 (green).

The materials have been processed to free-standing electrodes and characterized as symmetrical supercapacitors in three different electrolytes: 1 M Li_2_SO_4_ (AQ), 1 M TEA-BF_4_ (O) in acetonitrile (ACN) and EMIM-BF_4_ (IL). The supercapacitors show a rectangular CV shape in all three electrolytes (Figure S8, Supporting Information File 1). The CVs of the three carbon materials are exemplarily shown for the organic electrolyte (O) in Figure 4A and give hint to a purely capacitive energy storage mechanism due to the absence of peaks. Further electrochemical characterization data can be found in Supporting Information File 1 (Figures S9–S12). The specific capacitances for the different carbon materials measured in different electrolytes are calculated by galvanostatic charge–discharge curves (Table 2). In general, all carbon materials show a higher specific capacitance (*C*_spec._) in aqueous electrolyte, which can be attributed to the higher ion conductivity of aqueous electrolytes [72]. The highest value of *C*_spec._ was calculated for PUUPC-800-2 with 99 F·g^−1^, which is associated to the high specific surface area of this sample compared to PUPC-800-3 and PUUPC-800-1.

Regarding the loss of specific capacitance when increasing the specific current from 0.1 to 10 A·g^−1^, PUUPC-800-2 shows the best rate capability in the aqueous electrolyte (Figure 4B). This can be associated with the higher volume ratio of mesopores to micropores, since mesopores are acting as transport pores enabling a fast electrolyte ion mobility [73–74]. Interestingly, the rate capability for this material in organic and ionic liquid electrolytes, however, is worse compared to the other two materials. This can be attributed to a low electric conductivity of the electrodes accompanied with the lower ionic conductivity of such electrolytes. This causes a higher resistance of the whole device and results in a lower rate capability. The difference of the electrode conductivities is also shown in the Nyquist plot (Figure 4C). A higher nitrogen content reduces the electric conductivity of the electrodes and thus, does not have a beneficial influence on the supercapacitor performance.

## Conclusion

We introduced an upcycling process for plastic waste to produce N-doped carbon materials in a sustainable synthesis. Polyurethane waste serves as a carbon (and nitrogen) source and is converted via a mechanochemical pathway with K_2_CO_3_ and, optionally, urea. The mechanochemical approach does not require any solvent, has a short reaction and process time and is realized in a facile setup. By using different amounts of activation and doping reagents, we obtained optimized carbon materials offering excellent properties such as a high specific surface area of 2150 m^2^·g^−1^, and a total pore volume of 0.9 cm^3^·g^−1^ (PUUPC-800-1). These N-doped carbon materials performed similarly well as supercapacitors from commercial carbon materials such as YP-50F, showing a specific capacitance up to 99 F·g^−1^ in Li_2_SO_4_, as well as a stable performance in TEA-BF_4_ with 83 F·g^−1^. By the mechanochemical upcycling with additional urea, the rate capability of the supercapacitor was enhanced and the obtained device exhibits 80% of its capacitance at a high specific current of 10 A·g^−1^ in aqueous electrolyte. The broader intention would be to transfer the process presented here to other difficult-to-process polymer waste and thus be able to further counteract the generation of waste. The application possibilities of these materials could also be extended to other energy storage systems such as Li-ion batteries or waste-water purification, wherever materials with a high surface area and improved wettability are required.

## Experimental

### Synthesis of N-doped carbon

In a similar manner to [36,64], nitrogen-doped porous carbon materials were produced from polyurethane (PU) foam as the carbon source, urea (U) added as a supplementary nitrogen source and potassium carbonate (PC) added as an activation reagent. The nitrogen source and the activation reagent were used in different molar ratios (Table 3). The synthesis was carried out in a 45 mL zirconium oxide milling vessel with twenty-two 10 mm diameter zirconium oxide milling balls (3.19 g each). First, the sprayed polyurethane was milled for 10 min in a Fritsch Pulverisette 7 premium line planetary ball mill operating at a rotation speed of 600 rpm. After addition of the activation and doping reagents, the mixture was then milled in the same ball mill and vessel for 30 min and at a rotation speed of 800 rpm. The resulting polymer was pyrolyzed for one hour in argon at 800 °C with a heating rate of 150 °C·h^−1^ and afterwards purified with diluted HCl and water.

**Table 3:** Sample code and amounts of PU, urea and K_2_CO_3_.

sample code	PU / g	urea / g	K_2_CO_3_ / g

PUPC-1	3	0	1
PUPC-2	3	0	2
PUPC-3	3	0	3
PUUPC-1	3	1	3
PUUPC-2	3	2	3
PUUPC-3	3	3	3
PUUPC-4	3	3	2
PUUPC-5	3	3	1

### Characterization

Nitrogen physisorption measurements were performed with a Quadrasorb EVO/SI from Quantachrome Instruments at −196 °C. The samples were degassed before all measurements under vacuum at 150 °C for at least 24 h. The multi-point BET method was used to calculate the specific surface areas of the materials. For each sample, the relative pressure range is given at the corresponding location.

The calculation of the total pore volume was performed at a relative pressure of *p*/*p*_0_ = 0.95. Assuming slit and cylindrical pore geometry, the pore size distributions were calculated from the adsorption branch using quenched solid density functional theory (QSDFT) method incorporated into the ASiQwin analysis software (Quantachrome). Micropore volumes were calculated from the cumulative pore volumes at a diameter of 2 nm.

Water vapor adsorption measurements were carried out at 25 °C on an Autosorb iQ from Quantachrome Instruments after vacuum activation at 150 °C for at least 24 h. The total pore volume was calculated at relative pressure of *p*/*p*_0_ = 0.96 for each material.

Elemental analysis was carried out with a vario Micro cube from Elementar. The elemental composition of carbon, hydrogen, nitrogen and sulfur of all samples is the average of three measurements.

IR spectra were measured on a BRUKER Vertex 70 with a Specac Golden Gate ATR unit. A resolution of 2 cm^−1^ was utilized and the resulting spectra were treated with ATR correction by the OPUS 6.5 software. The spectra were recorded in the range of 4000–400 cm^−1^.

Electric powder conductivities were measured with an Agilent 34420A combined with a custom-built cell with a diameter of 1 cm. The powders were pressed with 2 t.

For the preparation of the electrodes, we added 5 wt % of polytetrafluoroethylene (PTFE granular, 98 wt % from Sigma-Aldrich) as binder to the N-doped carbon material, which was ground under heat treatment in a mortar. The resulting dough-like material was rolled out to a thickness of 100–200 µm and then cut out to a round electrode with a diameter of 10 mm. The electrodes were dried in a vacuum oven at 120 °C for 24 h.

For electrochemical testing, a specially manufactured polyether ether ketone (PEEK) cell with spring-loaded titanium pistons was used as symmetrical full cell, as described in detail elsewhere [75]. Electrode discs with a thickness of 100–200 µm and a diameter of 10 mm were punched out of the free-standing film electrode. Electrodes with the same mass were selected as the working and the counter electrode, which were placed on the current collector and separated by a glass-fiber separator (GF/A, Whatman). The prepared cells were filled with the electrolyte. We used a potentiostat/galvanostat VMP-3 from BioLogic for cyclic voltammetry (CV) and galvanostatic cycling with potential limitation (GCPL). CVs were recorded in full-cell mode at a scan rate 5 mV·s^−1^. In GCPL mode the specific current was increased from 0.1 to 10 A·g^−1^. In order to obtain information about the IR drop, a rest period of 10 s was introduced between charging and discharging.

The gravimetric capacitance was calculated from the discharge curve via the following equation:

[1] $$C_{\text{spec}}=\frac{4I}{\frac{\Delta U}{\Delta t}\cdot m}\;,$$

with specific capacitance *C*_spec_, cell voltage *U* corrected by IR drop, and carbon mass of both electrodes *m* (without binder).

## Supporting Information

File 1 Materials and methods, additional figures and activation mechanism.
