# Supplementary material for: Upcycling of polyurethane waste by mechanochemistry: synthesis of N-doped porous carbon materials for supercapacitor applications
Source: Beilstein J Nanotechnol. 2019 Aug 6;10:1618–27. doi: 10.3762/bjnano.10.157 (PMC6693373; doi:10.3762/bjnano.10.157)
Supplement: File 1 — Materials and methods, additional figures and activation mechanism. [file Beilstein_J_Nanotechnol-10-1618-s001.pdf]

BEILSTEIN JOURNAL OF NANOTECHNOLOGY

Supporting Information

for

Upcycling of polyurethane waste by mechanochemistry: synthesis of N-doped porous carbon materials for supercapacitor applications

Christina Schneidermann, Pascal Otto, Desirée Leistenschneider, Sven Grätz, Claudia Eßbach and Lars Borchardt

Beilstein J. Nanotechnol. **2019,** *10,* 1618–1627. doi:10.3762/bjnano.10.157

Materials and methods, additional figures and activation mechanism

License and Terms: This is a supporting information file under the terms of the Creative Commons Attribution License (http://creativecommons.org/licenses/by/4.0). Please note that the reuse, redistribution and reproduction in particular requires that the authors and source are credited.
The license is subject to the *Beilstein Journal of Nanotechnology* terms and conditions: (https://www.beilstein-journals.org/bjnano)

Contents

S1 Materials and Methods S3

S2 Figures S4

S2.1 IR spectra S4

S2.2 XRD S4

S2.3 SEM S5

S2.4 TGA S6

S2.5 Dynamic contact angle measurements S7

S2.6 Conductivity measurements S7

S2.6 Electrochemical characterization S8

S3 Activation mechanism S10

S1 Materials and Methods

The polyurethane foam was purchased from SOUDAL. Urea and K_2CO_3 were purchased from Grüssing GmbH. All chemicals were used without further purification.

Scanning electron microscopy (SEM) was carried out using a Hitachi SU 8020 at a beam voltage of 2 kV.

Thermogravimetric analysis (TGA) was performed on a Netzsch STA 409 PC/PG system using alumina crucibles under argon stream with a heating rate of 10 °C·min^{-1}.

X-ray diffraction (XRD) measurements were performed on a Xpert Pro from PANalytical using Cu Kα_1 (λ = 1.54056 Å) radiation as five-fold determination in the 2θ range of 5–70° with a step width of 0.026. The received X-ray diffractograms were compared with the Inorganic Crystal Structure Database (ICSD) regarding position and intensity of the reflections.

S2 Figures

S2.1 IR spectra

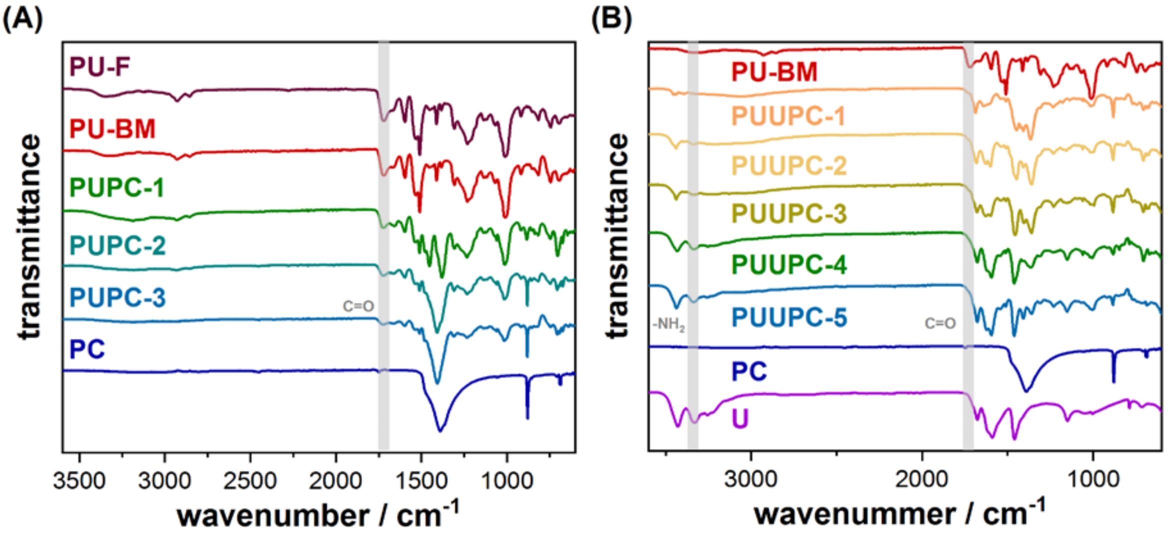

Figure S1: (A) Infrared-spectra of PU foam (PU-F) compared with PU ball-milled (PU-BM) with different amounts of K_2CO_3 (PUPC) and (B) PU-BM compared with PU milled with different amounts of K_2CO_3 and urea (PUUPC).

S2.2 XRD

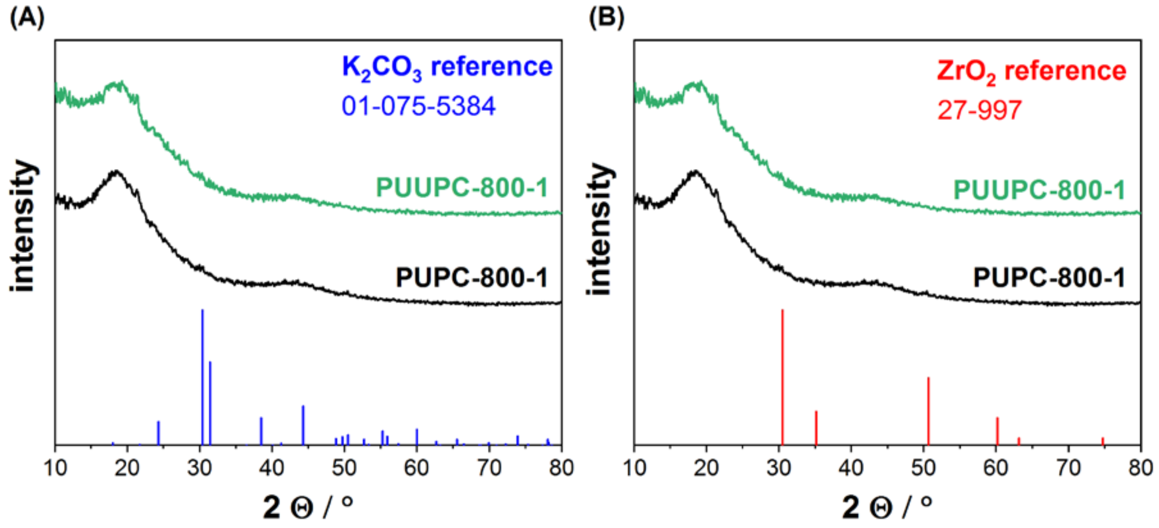

Figure S2: X-ray diffractograms of PUUPC-800-1 (green) and PUPC-800-1 (black) with K_2CO_3 reference (A/blue) and ZrO_2 reference (B/red).

S2.3 SEM

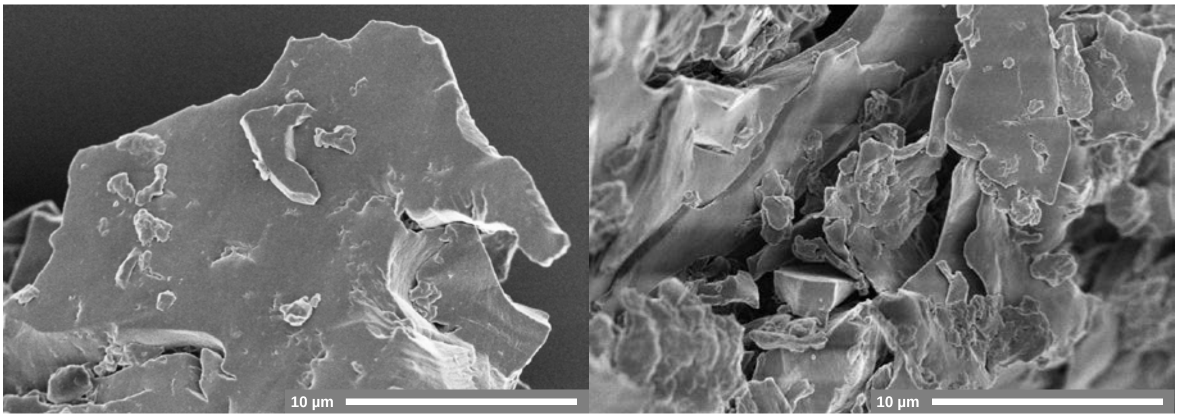

Figure S3: SEM images of PU-BM before pyrolysis.

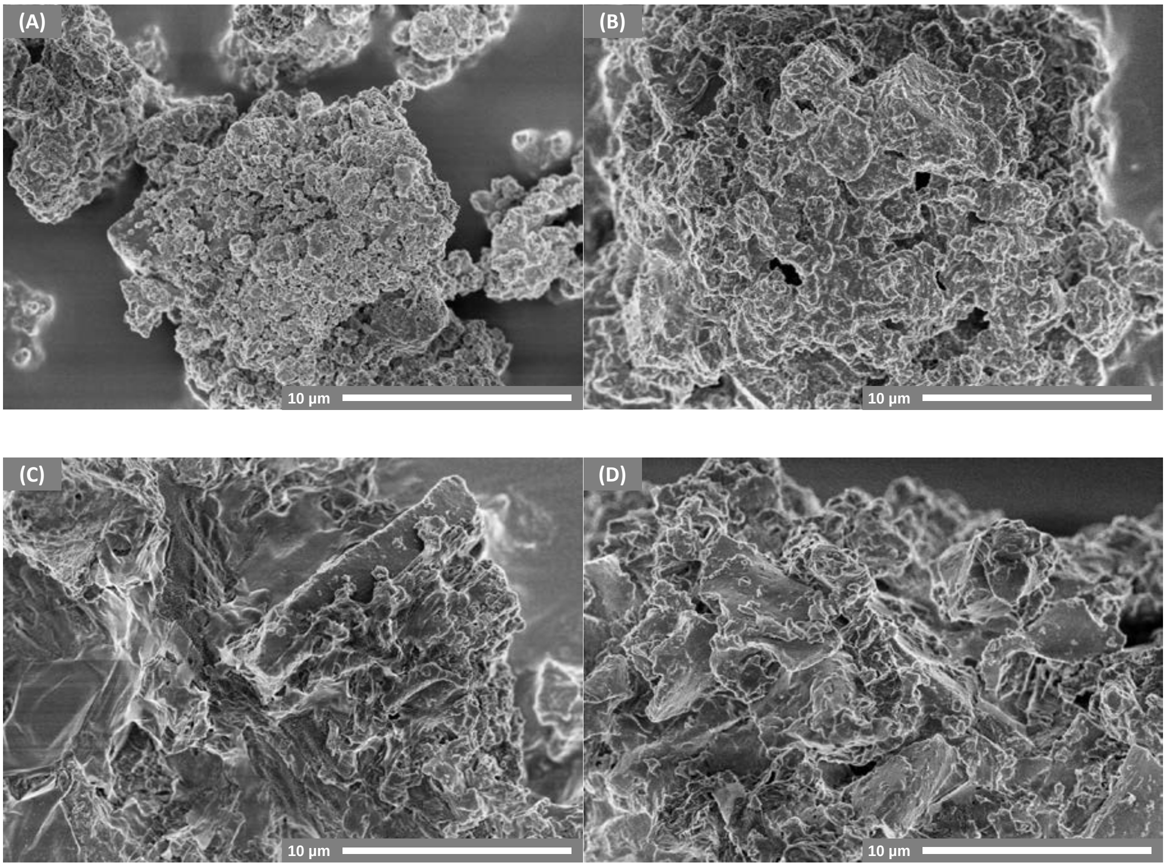

Figure S4: SEM pictures of PUPC-3 (A), PUUPC-1 (B), PUUPC-2 (C) and PUUPC-3 (D) before pyrolysis.

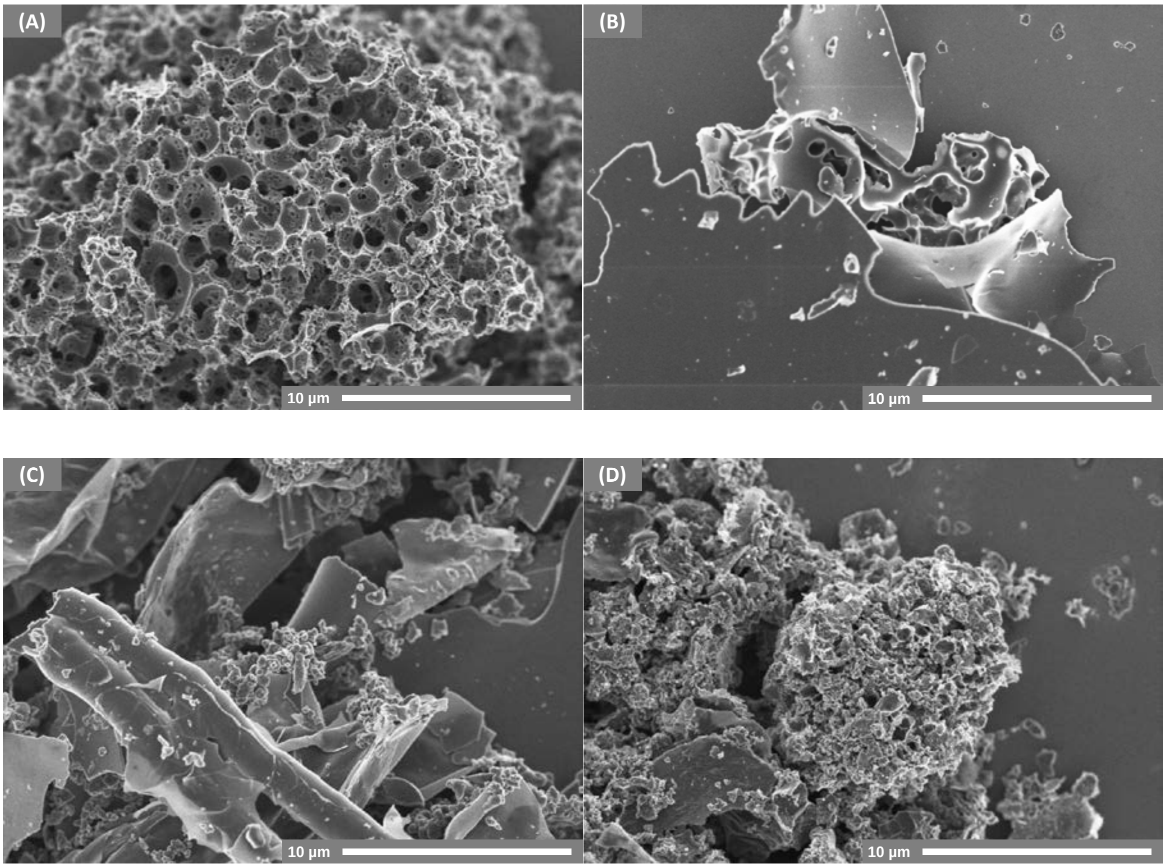

Figure S5: SEM pictures of the samples PUPC-3-800 (A), PUUPC-1-800 (B), PUUPC-2-800 (C) and PUUPC-3-800 (D).

S2.4 TGA

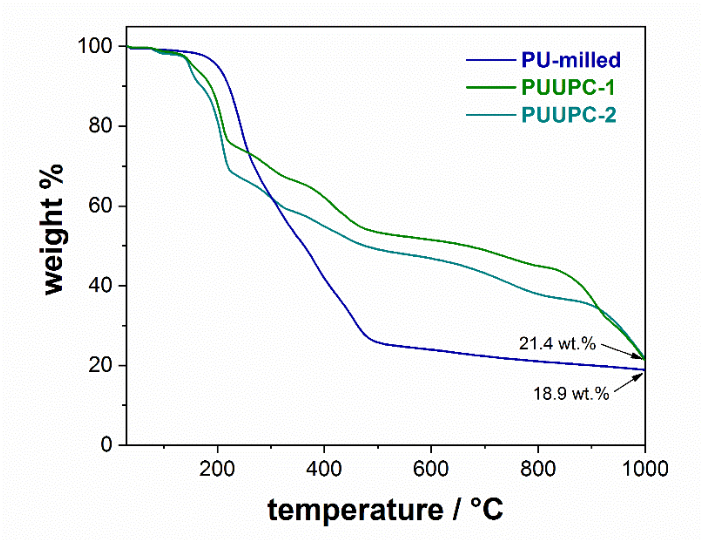

Figure S6: Thermogravimetric analysis of the samples PU-BM (blue), PUUPC-1 (green) and PUUPC-2 (cyan).

S2.5 Dynamic contact angle measurements

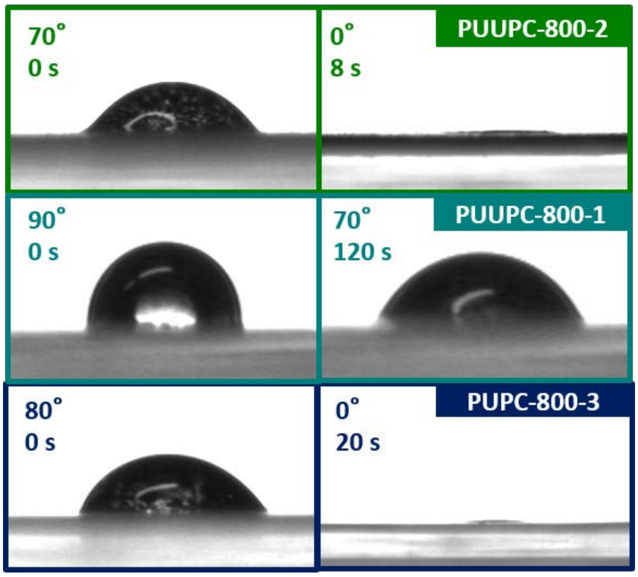

Figure S7: Dynamic contact angle measurements of the samples PUUPC-800-2 (green), PUUPC-800-1 (cyan) and PUPC-800-3 (blue).

S2.6 Conductivity measurements

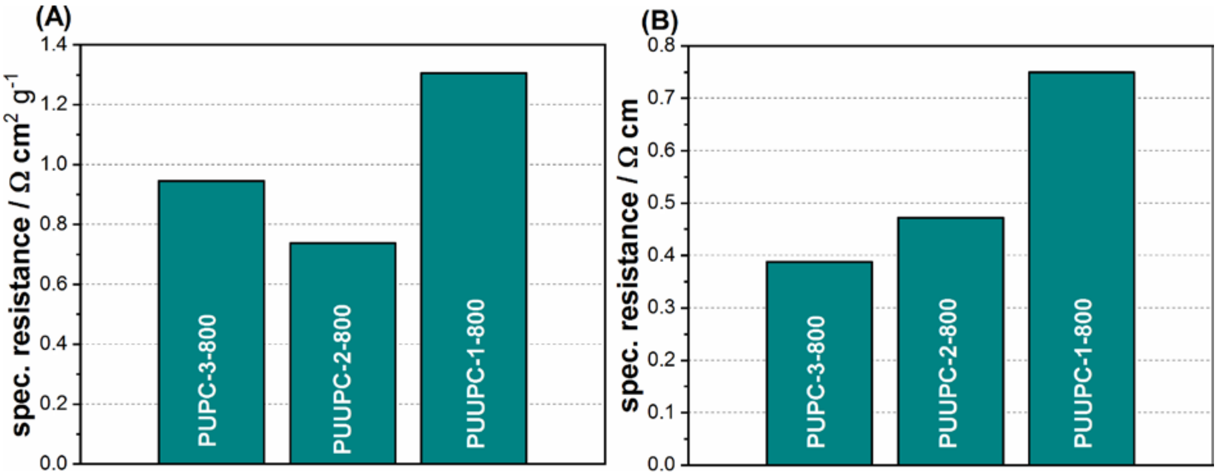

Figure S8: Powder conductivities. Gravimetric (A) and normalized (B) to the surface of electrode (d = 1 cm) for the samples PUPC-3-800, PUUPC-2-800 and PUUPC-1-800.

S2.6 Electrochemical characterization

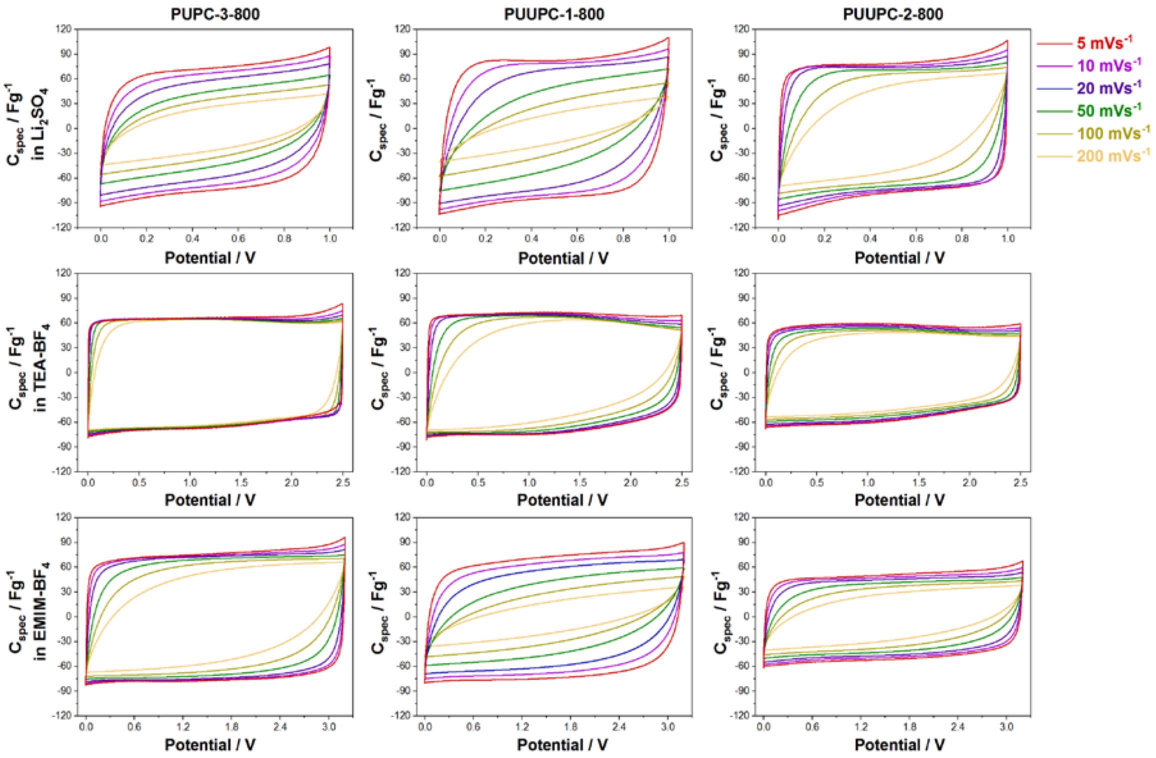

Figure S9: Cyclic voltammetry (CV) matrix of samples PUPC-3-800, PUUPC-1-800, and PUUPC-2-800 measured in aqueous electrolyte Li_2SO_4, organic electrolyte TEA-BF_4 in ACN and ionic electrolyte EMIM-BF_4.

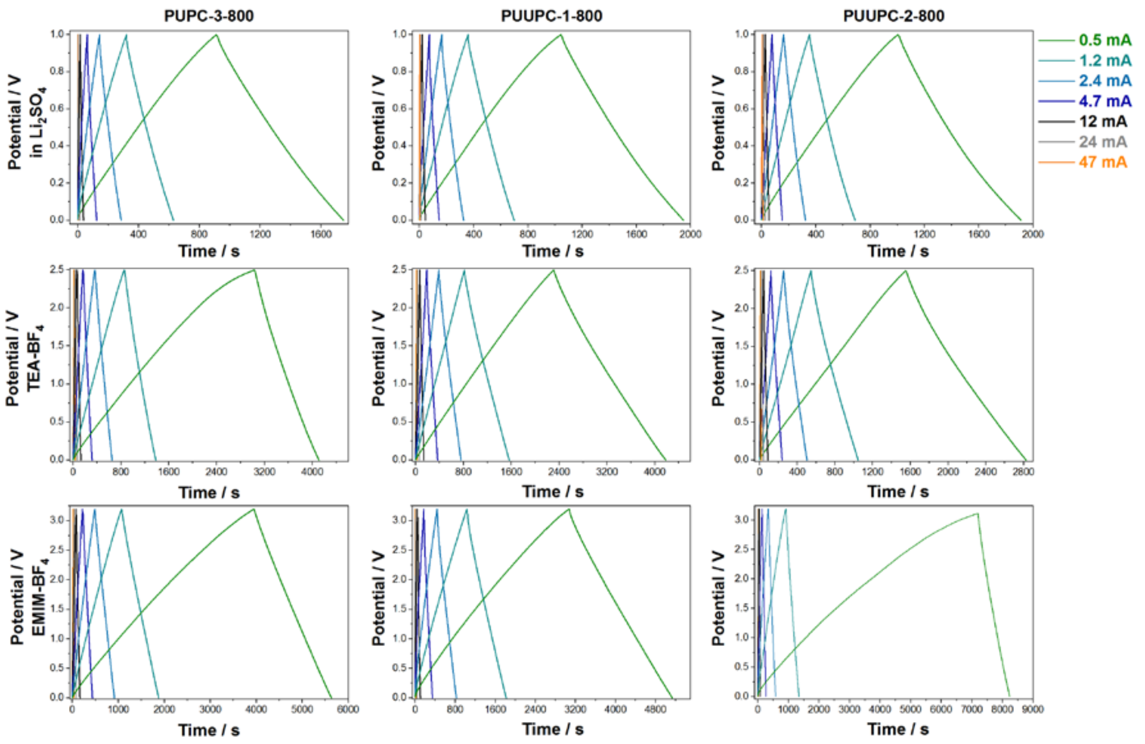

Figure S10: Galvanostatic cycling with potential limitation (GCPL) matrix of samples PUPC-3-800, PUUPC-1-800, and PUUPC-2-800 measured in aqueous electrolyte Li_2SO_4, organic electrolyte $TEA\text{-}BF_4$ in ACN and ionic electrolyte $EMIM\text{-}BF_4$.

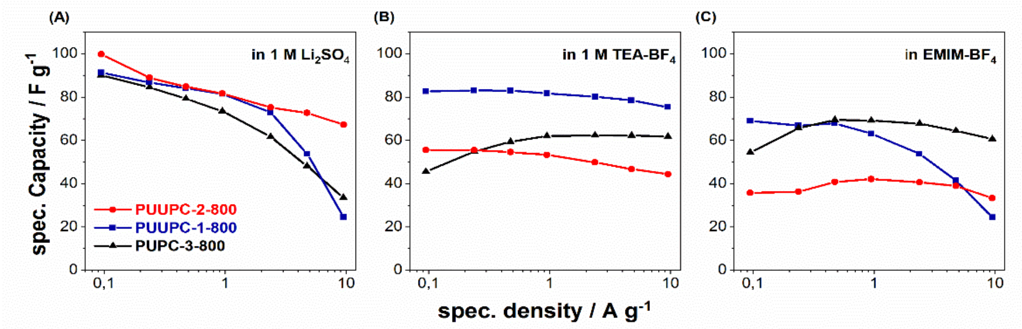

Figure S11: Galvanostatic charge–discharge rate handling plots at different specific current values of the samples PUUPC-800-1 (blue), PUUPC-800-2 (red) and PUPC-800-3 (black) in (A) aqueous Li_2SO_4 electrolyte, (B) organic $TEA\text{-}BF_4$ in ACN and (C) ionic liquid $EMIM\text{-}BF_4$.

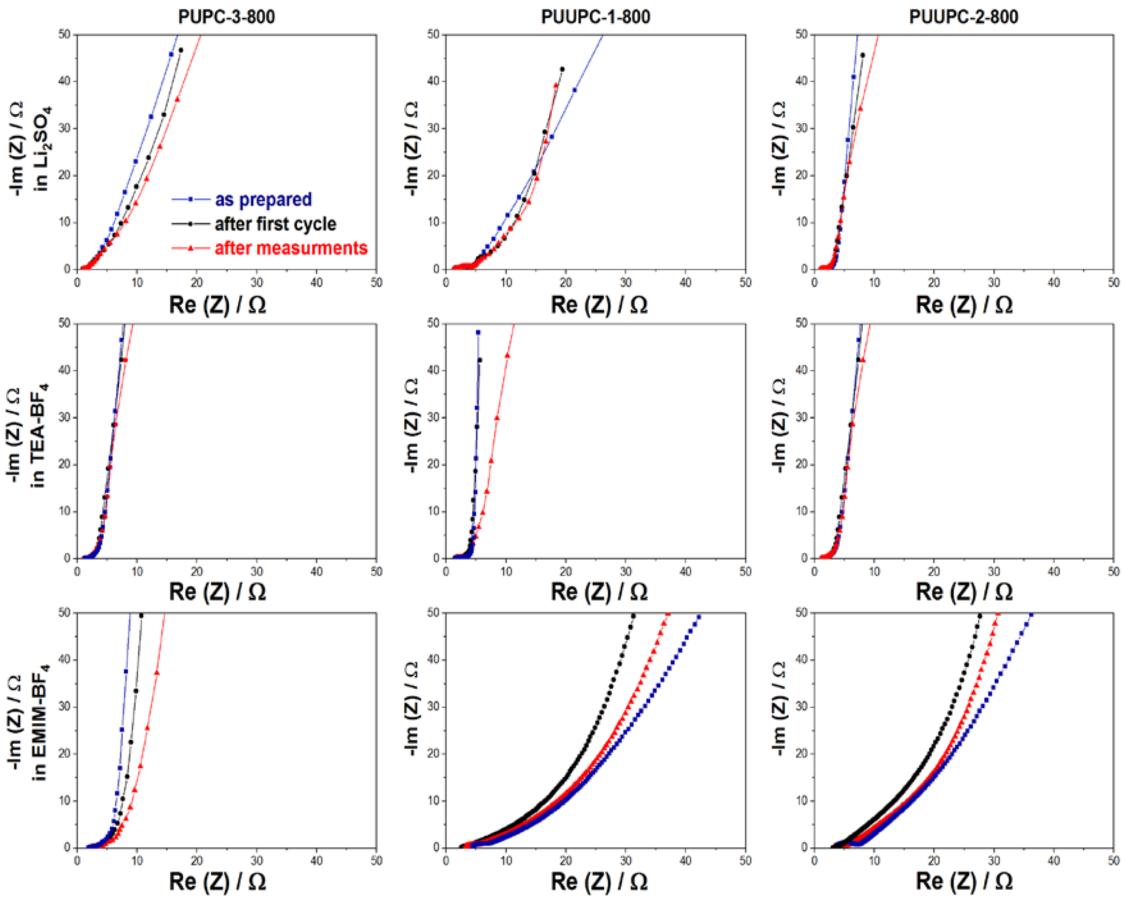

Figure S12: Potential electrochemical impedance spectroscopy (PEIS) matrix of samples PUPC-3-800, PUUPC-1-800, and PUUPC-2-800 measured in aqueous electrolyte Li_2SO_4, organic electrolyte TEA-BF_4 in ACN and ionic electrolyte EMIM-BF_4.

S3 Activation mechanism

Mechanism of activation with K_2CO_3 according to McKee et al. [66]:

$$K_2CO_3 + 2\,C \rightleftharpoons 2\,K + 3\,CO$$

$$2\,K + 2n\,C \rightleftharpoons 2\,C_nK$$

$$2\,C_nK + CO_2 \rightleftharpoons (2C_n)K_2O + CO$$

$$(2C_n)K_2O + CO_2 \rightleftharpoons 2\,nC + K_2CO_3$$
